# A common arrhythmia, not so common at an old age

**DOI:** 10.1007/s12471-013-0512-x

**Published:** 2014-01-10

**Authors:** A. A. M. Wilde

**Affiliations:** Academic Medical Centre, Amsterdam, Netherlands

Answer to rhythm puzzle

**Fig. 1:**
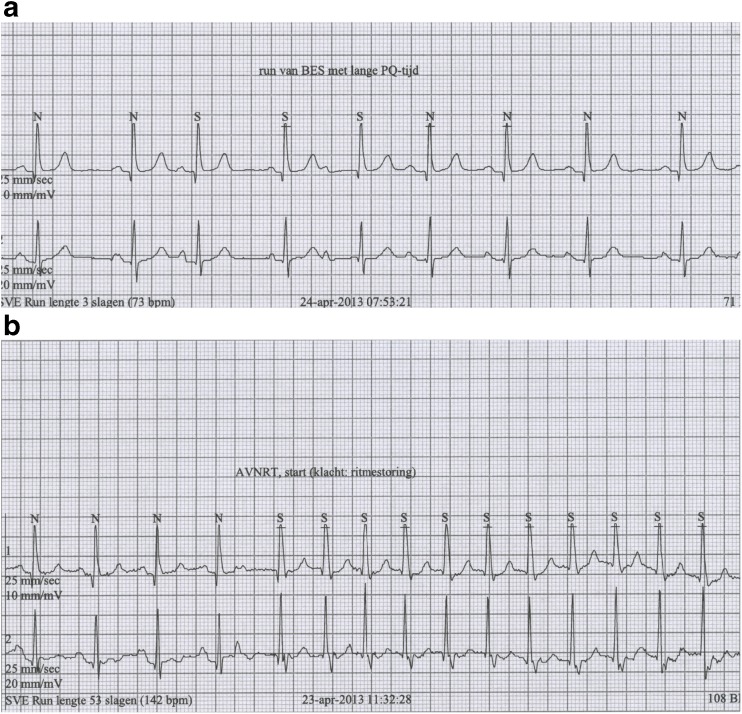
Figure 1a shows a two lead recording with a supraventricular rhythm that is most likely sinus rhythm (the upper lead is very likely lead 2 and there is a positive P wave in this lead). There are two P waves that come earlier and have a slightly different P-wave configuration (atrial extrasystoles). The 3rd P wave conducts to the ventricle with a normal (short) PR interval and the 5th P wave with a markedly prolonged PR interval. This suggests the presence of dual AV-nodal pathways. Figure 1b shows the onset of a supraventricular tachycardia (SVT), which is initiated by an atrial extrasystole that is visible early in the T wave of the 4th QRS complex. The SVT starts with a similarly slow PR conduction and clearly a retrograde P wave is visible immediately following the QRS complex (in particular in the lower lead). Now, due to an even slower conduction through the AV node (compared with Fig. 1a) there is the possibility of retrograde conduction to the atrium and an AV nodal re-entrant tachycardia (AVNRT) is initiated. This episode coincided with her complaints. Hence the correct diagnosis is AVNRT

